# Prolonged Grief-Related Symptoms Among Young Individuals After Loss of a Parent or Sibling to Cancer: A Systematic Review and Meta-Analysis

**DOI:** 10.3390/jcm15031060

**Published:** 2026-01-29

**Authors:** Chen Ee Low, Jia Yang Tan, Weiling Amanda Tan, Jayanth Jayabaskaran, Emily Chen Fei Ni, Ga Eun Pang, Dawn Yi Xin Lee, Sean Loke, Hon Jen Wong, Chun En Yau, Ainsley Ryan Yan Bin Lee, Cyrus Su Hui Ho

**Affiliations:** 1 Yong Loo Lin School of Medicine, National University of Singapore, Singapore 119228, Singapore; 2 Faculty of Medicine, University of Queensland, Brisbane, QLD 4102, Australia; 3 School of Medicine, Dentistry and Nursing, University of Glasgow, Glasgow G12 8QQ, UK; 4 Department of Psychological Medicine, Yong Loo Lin School of Medicine, National University of Singapore, Singapore 119228, Singapore; 5 Department of Psychological Medicine, National University Hospital, Singapore 119074, Singapore

**Keywords:** prolonged grief, grief, bereavement, neoplasm, cancer, young adults

## Abstract

**Background/Objectives:** Bereavement in childhood, adolescence, and young adulthood is associated with a range of grief responses, and a subset of bereaved individuals develop persistent or severe grief symptoms. Understanding the prevalence and risk factors of prolonged grief symptoms is important for guiding supportive care. **Methods:** We systematically searched PubMed, MedLine, Embase and PsycINFO for all studies comparing the prevalence and prognostic factors of prolonged grief-related symptoms among young individuals following parental or sibling death from cancer. Young individuals were defined as those not more than 25 years old before losing a parent or sibling to any cancer. Prolonged grief-related symptoms were defined as the presence of grief symptoms at least six months following the death of a parent or sibling of the bereaved person. Retrospective cross-sectional studies were included for evaluating prognostic factors affecting prolonged grief-related symptoms, but were not used for meta-analyses. Random-effects meta-analyses were conducted for the primary analysis. **Results:** From 1561 records identified, thirteen studies were included with five for quantitative pooling in meta-analysis. The pooled prevalence of self-reported prolonged grief-related symptoms was 48% (95% CI: 29–67%). Stratified analyses suggested a prevalence of 28% (95% CI: 7–65%) after parental death and 59% (95% CI: 45–72%) after sibling death. Factors associated with elevated prolonged grief-related symptoms included pre-existing depression, emotional difficulties, and insomnia. As no included studies conducted diagnostic clinical interviews, prolonged grief disorder according to the ICD-11 or DSM-5-TR criteria could not be assessed. **Conclusions:** Prolonged grief-related symptoms appear common among young individuals bereaved by loss of a parent or sibling to cancer, especially after sibling loss. However, interpretation remains limited by substantial heterogeneity, such as outcome measures, symptom thresholds, assessment time window, non-validated symptom measures, and predominance of cross-sectional studies. Future larger and methodologically rigorous studies using validated grief instruments across diverse settings are needed to clarify grief trajectories and guide developmentally appropriate intervention strategies.

## 1. Introduction

The loss of a parent or sibling to cancer is one of the most traumatic experiences a young individual can face in their lifetime, with a potential impact on their mental and physical well-being [1]. While grief is a universal human response to such a loss, there exists a proportion of these young individuals who experience prolonged grief. Prolonged grief disorder is characterised by intense and persistent grief that impairs daily functioning and causes significant distress for at least six months to a year of bereavement [2]. The impact of prolonged grief on young individuals involves many aspects of a young individual’s life, such as their physical health, emotional well-being, interpersonal relationships, and all-cause mortality [3].

The loss of a family member to cancer can be a distressing period for young individuals who are in their formative years [4]. They may have to endure a long period watching the progressive physical and mental deterioration of their loved ones [5,6]. During this period, there could also be shifting family roles as they take on caregiving duties that could be beyond their capacity [7]. Much of their energy would be on performing their caregiving duties well and managing the demands of taking care of a dying family member, hence leaving no room for these young individuals to process their emotions properly [7]. Such prolonged stressors may constrain opportunities for emotional processing, heighten vigilance or anxiety, and introduce chronic strain that may shape grief responses after the loss. Second, young individuals’ understanding of illness and death evolves with age, and periods of cognitive immaturity may limit comprehension of terminal illness or the finality of death [8,9]. This challenging experience can potentially increase their vulnerability to prolonged grief that persists beyond the loss. Identifying prolonged grief is crucial as traumatic events in early life may be associated with an increased vulnerability to developing psychological symptoms and disorders in later life [10,11,12,13,14].

Prior literature exploring prolonged grief-related symptoms among young individuals bereaved by cancer has reported widely varying prevalence estimates [15,16]. Such variation may reflect differences in study design, follow-up duration, cultural context, and measurement approaches. Some studies employ validated grief-specific questionnaires, while others use single items or broad indicators of distress. The absence of diagnostic interviews further complicates interpretation, as symptom thresholds may not correspond to clinical levels of impairment. Nevertheless, understanding prolonged grief-related symptom burden in this population is important for identifying risk factors, improving supportive care, and informing developmentally sensitive interventions.

To the authors’ best knowledge, this is the first systematic review and meta-analysis exploring the prevalence of prolonged grief-related symptoms in young individuals after a parental or sibling loss to cancer. Secondary outcomes include evaluating prognostic factors affecting prolonged grief-related symptoms. Understanding these factors is essential for providing appropriate support and care, as early intervention may help mitigate the long-term psychological consequences of grief in this vulnerable population.

## 2. Methods

We prospectively registered our protocol on PROSPERO (Reference: CRD42024539397). We followed the Preferred Reporting Items for Systematic Reviews and Meta-Analyses (PRISMA) guidelines [17], and the PRISMA Checklist was included in the Supplementary Materials.

### 2.1. Definitions

According to the World Health Organisation [18], we define young individuals as those not more than 25 years old at the time of losing a parent or sibling to any solid or haematological cancer. We defined prolonged grief disorder as persistent grief reactions after the death of a parent or sibling in the bereaved person for at least six months in accordance with ICD-11 or DSM-5-TR criteria [2]. “Prolonged grief-related symptoms” referred to self-reported symptoms of grief lasting 6 months or more post-bereavement, without a formal diagnosis of prolonged grief disorder (PGD) under ICD-11 or DSM-5-TR criteria. In our review, studies utilised questionnaires for self-reporting of prolonged grief-related symptoms, rather than diagnostic clinical interviews. As such, the prevalence of PGD was not assessed.

### 2.2. Search Strategy

A comprehensive literature search was conducted across PubMed, Embase, Cochrane, and PsycINFO. The strategy integrated search terms related to ‘Pediatrics’, ‘Child’, ‘Young Adults’, ‘Grief’, ‘Prolonged Grief’, ‘Bereavement’, ‘Neoplasm’, and ‘Cancer’. Controlled vocabulary specific to each database was utilised for subject heading searches, alongside a wide range of synonyms and relevant truncations for searches within titles, abstracts, and author keywords. The search strategy was adapted for each database. Detailed search strategies for PubMed and Embase are provided in Supplementary Table S1.

### 2.3. Inclusion and Exclusion Criteria

Two reviewers independently screened the titles and abstracts of all identified studies according to the inclusion and exclusion criteria. Studies assessed as ‘relevant’ or ‘unclear’ by the same two reviewers were resolved by consultation with a third independent reviewer.

**Figure 1:**
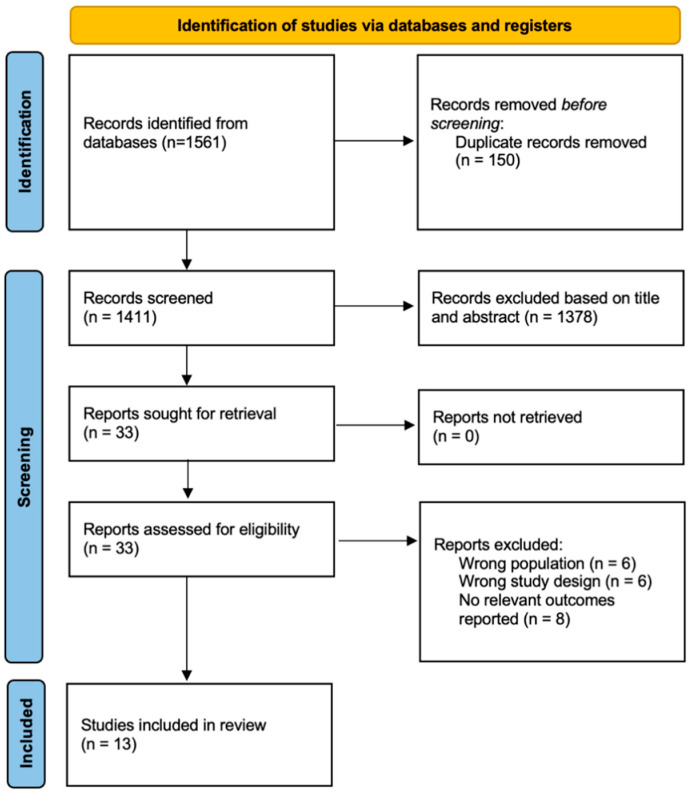
PRISMA Flowchart.

We included all English-language peer-reviewed studies published from database inception to 16 January 2026 that assessed the prevalence of prolonged grief-related symptoms among young individuals following parental or sibling death due to cancer. Non-empirical studies, case reports or series, conference abstracts and grey literature were excluded. We outlined the selection process in Figure 1.

### 2.4. Data Extraction and Analysis

Subject matter information extracted included demographics, previous medical history, the instruments and scales used to assess prolonged grief-related symptoms, duration since loss, and the main findings of the study. The number of participants and the number of events for prolonged grief-related symptoms were extracted to pool prevalence data. The instrument and method of assessing prolonged grief-related symptoms were extracted and reported.

We performed the analyses on R (version 4.1.0) with the *meta* and *metafor* packages. A two-sided *p* value of <0.05 was considered statistically significant. Under a generalised linear mixed model (GLMM), a meta-analysis of proportions was used to meta-analyse the prevalence of prolonged grief-related symptoms [19]. Studies from the same countries with overlapping populations were not included in the meta-analysis to prevent representation bias. We systematically evaluated potential overlaps between studies based on their data source (e.g., gathering data from the same national registry or databases). When two or more studies were determined to report data from the same population, only one study will be included in the meta-analysis. Selection of the retained studies prioritises the most comprehensive assessment of outcomes relevant to the review, the largest proportion or the most recent publication. The other overlapping studies were excluded from the meta-analysis but still retained for systematic review to evaluate for prognostic factors of prolonged grief-related symptoms. Details of the overlap assessment and study selection decisions are provided in Supplementary Table S2. We only performed subgroup analysis on the relationship with the deceased family member. Other subgroup analyses were not performed due to insufficient studies. As there were insufficient studies for subgroup analyses, prognostic factors affecting prolonged grief-related symptoms were evaluated using a synthesis without meta-analysis approach. Retrospective cross-sectional studies were included in the systematic review to evaluate potential prognostic factors that could affect prolonged grief-related symptoms, but were excluded from the prevalence meta-analysis because the number of events was equivalent to the total cohort size. Between-study heterogeneity was represented by I2 and τ2 statistics, with I2 of <30% as low heterogeneity, 30% to 60% as moderate heterogeneity, and >60% as substantial heterogeneity [20]. Sensitivity analyses were performed using influence diagnostics and heterogeneity exploration, including identification and exclusion of potential outliers and leave-one-out analyses.

### 2.5. Risk of Bias Assessment

Two independent reviewers assessed for quality and risk of bias of the studies using the Joanna Briggs Institute (JBI) Critical appraisal checklist for cross-sectional studies [21]. All discrepancies were resolved through the independent verdict of a third reviewer.

## 3. Results

From 1561 reports identified from the databases (Figure 1), we included a total of 13 studies [16,22,23,24,25,26,27,28,29,30,31,32,33] reporting the prevalence, incidence and risk factors of prolonged grief-related symptoms among young individuals following parent or sibling’s death from cancer. The remaining 1548 studies were not included after removing duplicates and irrelevant studies with the wrong population, study design and unrelated outcomes.

The characteristics of the 13 studies [16,22,23,24,25,26,27,28,29,30,31,32,33] are reported in Table 1. The number of participants included in each study ranged from 20 to 622, totaling 3231 participants in the final analysis. Only two studies used validated prolonged grief-related symptom measures (PG-13). The remaining studies used unvalidated or single-item measures capturing general grief reactions or related emotional distress [16,22,26,30,33]. Twelve of the studies [22,23,24,25,26,27,28,29,30,31,32,33] were from Sweden, and one study was from Norway [16]. Eight of the studies were cross-sectional [16,22,25,26,29,30,31,33] and five were retrospective cross-sectional studies [23,24,27,28,32]. Only five out of 13 studies measured and reported quantitative outcomes of prolonged grief-related symptoms amenable for quantitative pooling in meta-analysis [16,22,26,30,33]. The mean age of participants at the time of study ranged from 12.8 to 27.4 years. The mean age of participants at the time of loss ranged from 12.4 to 25 years. Eight studies [22,23,24,25,26,28,29,32] investigated prolonged grief-related symptoms among young individuals after parental death, while five studies [16,27,30,31,33] investigated prolonged grief-related symptoms among young individuals after a sibling’s death.

Meta-analysis was performed to evaluate the prevalence of self-reported prolonged grief-related symptoms in young individuals.

**Figure 2:**
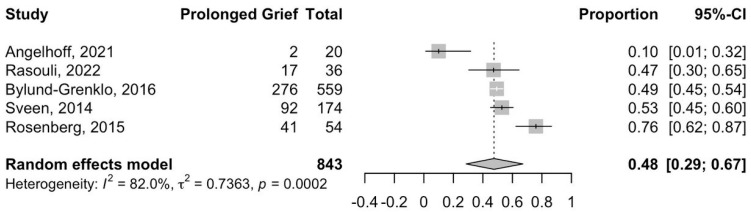
Prevalence of young individuals with self-reported prolonged grief-related symptoms.

The meta-analysis of five studies [16,22,26,30,33] (Figure 2) indicated that the prevalence of self-reported prolonged grief-related symptoms in young individuals is 48% (95% CI: 29–67%). Angelhoff et al. [22] studied prolonged grief-related symptoms among 20 adolescents with a mean age of 15.3 years for one to four years following the death of their parents from cancer. Rasouli et al. [16] explored the prevalence of unresolved grief and the impact of resilience and social support among 36 adolescents two to ten years after the loss of their sibling to cancer. Bylund-Grenklo et al. [26] looked at unresolved grief and its consequences among 559 adolescents six to nine years after the death of their parents from cancer. Sveen et al. [30] followed 174 young adults up for two to nine years after the death of their sibling from cancer. Rosenberg et al. [33] explored prolonged grief-related symptoms and their long-term psychological outcomes amongst 54 adolescents five to seventeen years after the loss of their siblings to cancer.

**Figure 3:**
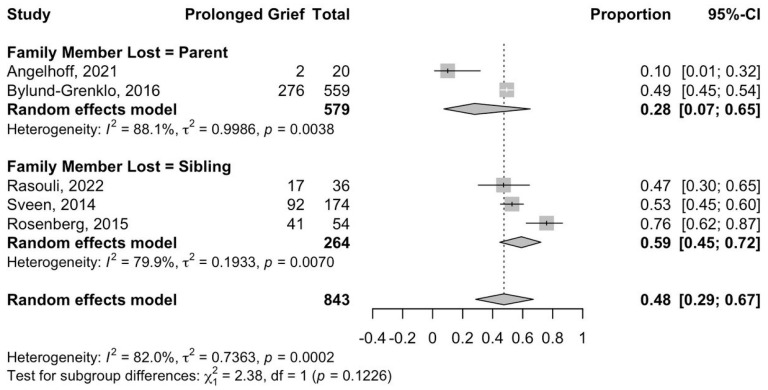
Prevalence of self-reported prolonged grief-related symptoms among young individuals stratified by relationship to the deceased family member.

Subgroup meta-analyses found that the prevalence of self-reported prolonged grief-related symptoms in young individuals after the death of a parent is 28% (95% CI: 7–65%) while the prevalence of self-reported prolonged grief-related symptoms in young individuals after the death of a sibling is 59% (95% CI: 45–72%) (Figure 3).

### 3.1. Systematic Review

Our study evaluated the prognostic factors affecting young individuals with prolonged grief-related symptoms. Poor prognostic factors were associated with pre-existing depression, pre-existing emotional problems, insomnia, gender, duration since loss and self-esteem.

### 3.2. Pre-Existing Depression

Three studies [26,27,31] explored the link between pre-existing depression and young individuals with prolonged grief-related symptoms (Supplementary Table S2). Both Bylund-Grenklo et al. and Lövgren et al. [26,31] found that having pre-existing depression was significantly associated with young individuals having prolonged grief-related symptoms, while Eilegård et al. [27] showed no significant association.

### 3.3. Pre-Existing Emotional Problems

Two studies [26,29] explored the association between having pre-existing emotional problems and young individuals with prolonged grief-related symptoms (Supplementary Table S3). Both studies found a significant association between pre-existing emotional problems and prolonged grief-related symptoms in young individuals. Bylund-Grenklo et al. [26] found that those with prolonged grief-related symptoms were significantly more likely to experience emotional numbness and self-injurious tendencies. Weber et al. [29] found that among those with prolonged grief-related symptoms, those aged 4 to 11 years were significantly more likely to have emotional problems, while adolescents aged 12 to 20 years were significantly more likely to have both emotional and conduct problems.

### 3.4. Insomnia

Two studies [26,27] investigated the association between insomnia among young individuals with prolonged grief-related symptoms (Supplementary Table S4). Both Bylund-Grenklo and Eilegård et al. [26,27] found that participants with prolonged grief-related symptoms were significantly more likely to experience insomnia. Bylund-Grenklo et al. [26] focused on exploring unresolved grief among 559 adolescents six to nine years after the death of their parents. In comparison, Eilegård et al. [27] followed up on 240 adolescents two to nine years after the death of their siblings.

### 3.5. Gender

Three studies [16,22,26] investigated the link between gender and having prolonged grief-related symptoms (Supplementary Table S5). Both Angelhoff and Rasouli et al. [16,22] found no significant association between gender and prolonged grief-related symptoms. Interestingly, only Bylund-Grenklo et al. [26] found a significant association between being female and experiencing prolonged grief-related symptoms compared to being male. The study investigators hypothesised that this is likely due to higher levels of exhaustion, sedative use, and dizziness among females, which may have hindered their ability to process their loss.

### 3.6. Duration Since Loss

Two studies [22,31] evaluated the association between the duration since the loss of their loved ones and prolonged grief-related symptoms in young individuals (Supplementary Table S6). Lövgren et al. [31] found that the time since death of two to four years and five to seven years was significantly associated with prolonged grief-related symptoms, while Angelhoff et al. [22] found no significant association.

### 3.7. Self-Esteem

Two studies [22,27] explored the link between the self-esteem of young individuals and those with prolonged grief-related symptoms (Supplementary Table S7). Eilegård et al. [27] found that low self-esteem was significantly higher in those with prolonged grief-related symptoms compared to their peers, while Angelhoff et al. [22] found no significant association.

### 3.8. Risk-of-Bias

The quality of the 13 studies [16,22,23,24,25,26,27,28,29,30,31,32,33] was assessed using the JBI tool for cross-sectional studies and presented in Supplementary Table S8. Across the domains assessed by the JBI risk-of-bias tool, all included studies were rated as having an overall low risk of bias.

### 3.9. Sensitivity Analyses

Identification and exclusion of potential outliers and leave-one-out analyses highlighted Angelhoff et al. [22] as an outlier (Supplementary Figures S1 and S2). This could possibly be due to the higher quality of parent-adolescent communication, likely fostering healthier coping mechanisms and emotional validation, enabling the adolescents to process their grief more effectively.

## 4. Discussion

To our knowledge, this study is the first systematic review and meta-analysis conducted on the prevalence of prolonged grief-related symptoms in young individuals following the loss of a parent or sibling to cancer. We found an elevated prevalence of self-reported prolonged grief-related symptoms of 48% (95% CI: 29–67%) in young individuals following the loss of a parent or sibling to cancer. Notably, our subgroup analysis showed that young individuals who lost a sibling to cancer had a higher prevalence of self-reported prolonged grief-related symptoms at 59% (95% CI: 45–72%), compared to those who lost a parent to cancer at 28% (95% CI: 7–65%). Our systematic review also identified pre-existing depression, pre-existing emotional problems and insomnia to be associated with the risk of prolonged grief-related symptoms among young individuals. While the pooled prevalence estimate of 48% suggests substantial grief-related difficulties, interpretation requires caution. The prevalence reflects self-reported elevated symptoms on heterogeneous tools, many of which were not designed to assess prolonged grief as defined in contemporary diagnostic systems. The wide confidence intervals, particularly in subgroup analyses, further reflect limited sample sizes and substantial heterogeneity across studies.

We compared our pooled estimate with existing literature on the prevalence of prolonged grief-related symptoms among youths who have lost a parent or sibling due to other causes of death (Table 2). However, direct comparison across the studies must be interpreted cautiously because these studies vary in outcome measurement encompassing prolonged grief disorder, prolonged grief-related symptoms and complicated grief symptoms. The studies also relied on self-reported symptom thresholds, rather than clinician-confirmed prolonged grief disorder. Within these constraints, our review found that young individuals who have lost a parent or sibling to cancer experienced a higher incidence of prolonged grief-related symptoms compared to those who have lost their family members to other causes. Studies in the literature reported that the prevalence of self-reported prolonged grief-related symptoms ranges from 12.9 to 19.7% among young individuals who have lost their parents to other causes such as suicide, accidents, natural causes and natural disasters. This is lower than our finding of 28% prevalence of self-reported prolonged grief-related symptoms for parental loss to cancer. Similarly, our finding of self-reported prolonged grief-related symptom prevalence of 59% for sibling loss to cancer is higher than the 16.1 to 29.4% range reported in other studies of sibling loss to other causes. Other than possible confounding due to bias in self-reporting and sampling in included studies, there may be several contributing factors. This difference could be due to the unique nature of cancer-related deaths [34,35]. Firstly, the diagnosis of cancer is often made a period of time before death occurs. The long disease course means a greater amount of time young individuals spend witnessing the gradual physical and psychological decline of their family member, which leads to anticipatory grief [36], complicating the grieving process [37]. Secondly, after a cancer diagnosis, there may be a significant sociodemographic impact on family dynamics, such as disruption of the way of life and role changes that can bring about more pressure and emotional stress to family members, adding more layers to their grief experience [38,39].

Young individuals who lost a sibling to cancer experienced a higher prevalence of prolonged grief-related symptoms compared to those who lost a parent, which is likely due to the unique nature of the sibling relationship. The sibling relationship is unique compared to the parent–child relationship, as it is expected that their relationships with their siblings will last longer throughout their lives. Therefore, the loss of a sibling can trigger an intense loss experience [45]. Furthermore, a study on bereavement among South African youths who lost their siblings to acquired immunodeficiency syndrome found that parents often provided inadequate emotional support and discouraged open expressions of grief [46]. Family cohesion following the loss of a sibling plays a crucial role in a bereaved child’s ability to cope with grief, and when the support is lacking, the child may feel distressed and forgotten [47]. Additionally, multiple studies have shown that parents experiencing their grief were less able to support their children emotionally, and their emotional unavailability can make emotional processing harder for the child [48,49]. Hence, prolonged grief-related symptoms can be worse in young individuals who have lost their sibling to cancer compared to their parents, due to the lack of emotional support from their parents and their inability to find emotional comfort from their family. Our results should be interpreted with prudence as the estimates were accompanied by wide confidence intervals, possibly reflecting limited statistical power and high heterogeneity.

Pre-existing depression was shown to be associated with higher rates of prolonged grief-related symptoms among young individuals. This is unsurprising, as there has been substantial evidence of how a history of depression can cause prolonged grief-related symptoms. For example, a longitudinal study conducted in the United States of the grief trajectories of 182 young individuals found that those with prolonged grief had higher rates of previous depression [50]. Additionally, individuals with previously diagnosed depression before experiencing loss had higher rates of complicated grief [51] and more distressing grief symptoms [52]. Depression and grief have been shown to both involve similar neurobiological alterations in the amygdala and prefrontal cortex, which are involved in emotional control and processing [53]. Hence, pre-existing depression can disrupt these neurological pathways, making these young individuals more prone to grief-related emotional stressors [54]. Furthermore, depression is associated with significant behavioural changes, including social isolation and anhedonia [55], which hinders healthy grief processing, as individuals would likely avoid social support and emotional regulation strategies [56]. Thirdly, negative thinking patterns, such as catastrophising and overgeneralisation [57], which are common in individuals with depression, can worsen their emotional psyche and maladaptive beliefs about themselves and the world, hindering their emotional processing as well [58,59]. Therefore, more proactive and regular follow-ups with young individuals in a bereaved family are recommended to actively monitor their emotional well-being, enabling early identification and timely intervention for those at heightened risk of prolonged grief-related symptoms.

Our study also found that having pre-existing emotional problems is significantly associated with prolonged grief-related symptoms. Grief inherently requires young individuals to process a wide range of emotions, which becomes particularly challenging when they are already struggling with emotional regulation [60,61]. Therefore, they might lack the needed emotional tools to regulate their grief when confronted with the loss of a family member [62]. Healthy grief processing requires individuals to be able to cope with emotional discomfort [63], and young individuals with emotional problems often use avoidance strategies to run away from this discomfort and thus prevent healthy grief processing [64]. As young individuals are in their early stages of emotional development, they are usually less capable than adults to cope with the death of a family member, especially if they have not had the chance to process their emotions during their parent’s illness [65]. Especially in cases where death is unexpected or a child’s distress is not addressed appropriately, they may struggle with emotion regulation and develop more complicated grief reactions, thereby contributing to prolonged grief-related symptoms. By highlighting this psychological burden, further studies may seek to develop strategies to mitigate the development and progression of prolonged grief. For example, young individuals who are identified to have poor emotion regulation strategies may be taught strategies on distress tolerance [66,67,68] and dialectical behavioural therapy techniques, which have demonstrated strong efficacy for improving emotion regulation in these young individuals [69].

Insomnia was also found to be associated with individuals having prolonged grief-related symptoms. Sleep disturbances impair emotional regulation [70], particularly in young individuals for whom slow-wave sleep is essential for emotional processing [71]. Since sleep plays a critical role in processing emotions and memories, individuals experiencing insomnia may struggle to process their grief, leading to difficulty in reaching emotional resolution and increasing their vulnerability to developing prolonged grief-related symptoms. Improvements in sleep would also reflect broader emotional healing, reduced hyperarousal, and better coping mechanisms. With actigraphy wearables becoming more commonplace, sleep tracking is a practical option for monitoring treatment progress over time and detecting early signs of relapse or ongoing distress. Cognitive Behavioural Therapy for Insomnia (CBT-I) has demonstrated effectiveness in treating insomnia and other comorbid mental disorders [72]. CBT-I could be adapted to include grief-specific content, supporting bereaved individuals to manage intrusive thoughts at night, reframe unhelpful beliefs related to sleep and loss, and establish healthy bedtime routines [72]. Thus, targeting insomnia through CBT-I could provide a promising psychotherapeutic approach towards treating prolonged grief-related symptoms.

### Limitations

Our study should be interpreted given certain limitations. Most significantly, there was heterogeneity and limited validity of measurement tools used across studies. Only two studies employed validated prolonged grief scales (PG-13), and none used clinician-administered diagnostic interviews. The majority used single items or study-specific questions that were not designed to assess prolonged grief disorder as defined in ICD-11 or DSM-5-TR [73]. Consequently, the pooled prevalence reflects elevated self-reported prolonged grief-related symptoms rather than clinically assessed PGD. Symptom thresholds varied widely, and in some cases, it was unclear whether cutoffs indicated distress, impairment, or both. This inconsistency, along with the lack of validation of instruments to assess prolonged grief-related symptoms, may inflate prevalence estimates, limit comparability, and obscure distinctions between developmentally normative grief responses and those indicative of persisting difficulties. As most studies used study-specific instruments that were self-reported, a higher rate of social desirability bias has to be considered. Young individuals may also interpret questionnaire items differently based on developmental level, cultural background, or personal understanding of grief. Second, the predominance of cross-sectional designs limits the ability to understand symptom trajectories, distinguish transient from persistent symptoms, or assess causal relationships. Longitudinal studies are needed to clarify how grief evolves over time and to identify which early symptoms predict later difficulties. Third, most studies relied on convenience or registry-based sampling within specific health systems. This may bias samples toward those engaged with healthcare services or those who remain contactable over time. It may also introduce selection bias as individuals with significantly increased symptoms may have had a higher likelihood of participating voluntarily in studies, resulting in the high prevalence of symptoms studied. Fourth, many studies did not comprehensively assess or adjust for potential confounders such as socioeconomic status, family functioning and social support, pre-loss mental health, or exposure to additional stressors. Fifth, given the broad developmental span (children through young adults up to age 25 years), interpretation of pooled prevalence estimates requires additional nuanced interpretation. Grief presentation, functional impact, and symptom measurement may differ across developmental stages. Younger children are more likely to express distress through behavioural or somatic symptoms, while adolescents and young adults may experience grief alongside disruptions to identity formation, autonomy, peer relationships, educational and vocational roles. Sixth, there was limited reporting of disease-related factors such as cancer trajectory, symptom burden and the relationship between the individual and family member that may shape bereavement outcomes. Without these contextual data, it is difficult to understand the mechanisms linking cancer-related experiences to later grief symptoms. This limits the granularity of subgroup analyses that could be performed. Lastly, there is also potential for residual confounding in our results due to several important contextual factors that were not consistently assessed across the included studies. These include family functioning, socioeconomic status, and illness trajectory. Such factors may meaningfully influence the bereavement process and the development and course of grief responses. Future studies should incorporate these contextual and illness-related variables to better elucidate the mechanisms underlying prolonged grief-related symptoms in bereaved young individuals.

## 5. Conclusions

Prolonged grief in young individuals following a parent’s or sibling’s death from cancer is an underexplored area in child psychiatry. We found an elevated pooled prevalence of self-reported prolonged grief-related symptoms in young individuals following the loss of a parent or sibling to cancer. Subgroup analyses showed that young individuals who lost a sibling to cancer had a two times higher prevalence of prolonged grief-related symptoms compared to those who lost a parent to cancer. Reported associations included pre-existing emotional issues, depression and insomnia. However, these findings should be interpreted cautiously, given substantial heterogeneity in outcome measurement, symptom thresholds, assessment time windows, possibility of errors or anomalies in data extraction or outcome reporting across studies, reliance on non-validated symptom instruments, cross-sectional study designs, and limited cultural diversity. There is an urgent need for methodologically rigorous, longitudinal, and culturally diverse research employing validated grief instruments and developmentally sensitive frameworks. Such work is essential for clarifying the natural history of grief symptoms, identifying young people at highest risk for persistent difficulties, and guiding targeted, early, and developmentally appropriate interventions.

## Figures and Tables

**Table 1:** Main characteristics of the included studies.

Author, Year	Study Type	Region of Study	Gender Male (Proportion 0–1)	Mean (SD) Age of Young Individuals (at Time of Study)	Mean Age (SD) of Young Individuals (During the Loss)	Mean (SD) Number of Years Since Loss	Who Was the Loved One Lost?	Post-Bereavement Time to Assessment (Years)	Scale Used to Assess for Prolonged Grief-Related Symptoms	Total Number of Participants	Number of Participants with Prolonged Grief-Related Symptoms
Angelhoff 2021	Cross-sectional	Sweden	0.4	15.3 (2)	12.4 (2)	3.4 (1)	Parents	Assessed 1–4 years after death	PG-13	20	2
Bylund-Grenklo 2021	Cross-sectional	Sweden	0.5	22 (NR)	NR (NR)	6–9 (NR)	Parents	Assessed 6–9 years after death	Study-specific questionnaire	622	280
Bylund-Grenklo 2016	Cross-sectional	Sweden	0.5	22 (NR)	14.6 (NR)	6–9 (NR)	Parents	Assessed 6–9 years after death	Study-specific questionnaire	559	274
Weber 2021	Cross-sectional	Sweden	0.55	12.78 (4.42)	NR (NR)	2.78 (0.78)	Parents	NR	PG-13	NR	NR
Sveen 2014	Cross-sectional	Sweden	0.42	24 (3.8)	17.7 (3.7)	6.3 (2.3)	Sibling	Assessed 2–9 years after death	Study-specific questionnaire	174	152
Lövgren 2018	Cross-sectional	Sweden	0.42	24 (3.8)	18 (3.7)	6 (2.3)	Sibling	Assessed 2–9 years after death	Study-specific questionnaire	148	79
Rasouli 2022	Cross-sectional	Norway	0.3	22.6 (2.3)	15.9 (2.5)	6.9 (2.4)	Sibling	Assessed 2–10 years after death	Study-specific questionnaire	36	17
Rosenburg 2015	Cross-sectional	Sweden	0.31	25.6 (7.8)	13.8 (7.3)	11.8 (3.2)	Sibling	Assessed 5–17 years after death	Study-specific questionnaire	54	41
Beernaert 2017	Retrospective cross-sectional	Sweden	0.5	22 (NR)	NR (NR)	6–9 (NR)	Parents	Assessed 6–9 years after death	Study-specific questionnaire	593	593
Birgisdóttir 2023	Retrospective cross-sectional	Sweden	0.49	NR (NR)	14.6 (NR)	6–9 (NR)	Parents	Assessed 6–9 years after death	Study-specific questionnaire	622	622
Eilegård 2013	Retrospective cross-sectional	Sweden	0.42	27.4 (NR)	NR (NR)	0.25–1 (NR)	Sibling	Assessed 2–9 years after death	Study-specific questionnaire	174	174
Lundberg 2020	Retrospective cross-sectional	Sweden	0.16	24 (NR)	NR (NR)	1.5 (NR)	Parents	Assessed 14–18 months after death	Study-specific questionnaire	55	55
Wallin 2016	Retrospective cross-sectional	Sweden	0.42	24.2 (NR)	12–25 (NR)	3.4 (1)	Parents	Assessed 2–9 years after death	Study-specific questionnaire	174	174

Abbreviations: NR, not reported; SD, standard deviation; PG, prolonged grief.

**Table 2:** Comparison of prolonged grief among different populations.

Author	Year	Country	Characteristics of the Study	Study Specific Instrument to Assess Prolonged Grief	Prevalence of Prolonged Grief
Brent [40]	2009	USA	Youth who lost a parent to suicide Youth who lost a parent to an accident Youth who lost a parent to natural causes	Inventory of Complicated Grief (ICG)	12.9% 16.2% 14.5%
Ozdemir [41]	2025	Turkey	Youth who lost a parent to an earthquake	Prolonged Grief Assessment-Child Version (PGA-C)	19.7%
Morris [42]	2016	USA	Youths who lost a sibling to medical illness, accident, homicide and suicide	Prolonged Grief-13 (PG-13)	16.1%
Thieleman [43]	2023	USA	Youths who lost a sibling to substance overdose, suicide, and accidental	Prolonged Grief-13-Revised (PG-13-R)	29.4%
Titlestad [44]	2024	Norway	Youths who lost a sibling to drug abuse	Special Grief Questions (SGQ)	21.8%

## Data Availability

The original contributions presented in this study are included in the article/Supplementary Materials. Further inquiries can be directed to the corresponding author.

## Supplementary Materials

The following supporting information can be downloaded at: https://www.mdpi.com/article/10.3390/jcm15031060/s1, Table S1: Search strategy; Table S2: De-duplication Workflow for Overlapping Cohorts and Final Study Selection; Table S3: Evaluation of the mediating or confounding effect of pre-existing depression in young individuals with prolonged grief-related symptoms; Table S4: Evaluation of the mediating or confounding effect of emotional problems in young individuals with prolonged grief-related symptoms; Table S5: Evaluation of the mediating or confounding effect of insomnia in young individuals with prolonged grief-related symptoms; Table S6: Evaluation of the mediating or confounding effect of gender in young individuals with prolonged grief-related symptoms; Table S7: Evaluation of the mediating or confounding effect of the duration since loss in young individuals with prolonged grief-related symptoms; Table S8: Evaluation of the mediating or confounding effect of self-esteem in young individuals with prolonged grief-related symptoms; Table S9: Quality assessment of included cross-sectional studies using the Joanna Brigg’s Institute Critical Appraisal tool; Figure S1: Leave-one-out analyses of studies assessing the proportion of young individuals with prolonged grief-related symptoms; Figure S2: Outlier assessment of studies assessing the proportion of young individuals with prolonged grief-related symptoms; PRISMA Checklist.
