# Supplementary material for: Prolonged Grief-Related Symptoms Among Young Individuals After Loss of a Parent or Sibling to Cancer: A Systematic Review and Meta-Analysis
Source: J Clin Med. 2026 Jan 29;15(3):1060. doi: 10.3390/jcm15031060 (PMC12898307; doi:10.3390/jcm15031060)
Supplement: Supplementary file 1 [file jcm-15-01060-s001.zip › jcm-4098760-supplementary.pdf]

Prolonged grief-related symptoms among young individuals after loss of a parent or sibling to cancer: A systematic review and meta-analysis

Supplementary Table S1: Search Strategy

Supplementary Table S2: De-duplication Workflow for Overlapping Cohorts and Final Study Selection

Supplementary Table S3: Evaluation of the mediating or confounding effect of pre-existing depression in young individuals with prolonged grief-related symptoms

Supplementary Table S4: Evaluation of the mediating or confounding effect of emotional problems in young individuals with prolonged grief-related symptoms

Supplementary Table S5: Evaluation of the mediating or confounding effect of insomnia in young individuals with prolonged grief-related symptoms

Supplementary Table S6: Evaluation of the mediating or confounding effect of gender in young individuals with prolonged grief-related symptoms

Supplementary Table S7: Evaluation of the mediating or confounding effect of the duration since loss in young individuals with prolonged grief-related symptoms

Supplementary Table S8: Evaluation of the mediating or confounding effect of self-esteem in young individuals with prolonged grief-related symptoms

Supplementary Table S9: Quality assessment of included cross-sectional studies using the Joanna Brigg's Institute Critical Appraisal tool

Supplementary Figure S1: Leave-one-out analyses of studies assessing the proportion of young individuals with prolonged grief-related symptoms

Supplementary Figure S2: Outlier assessment of studies assessing the proportion of young individuals with prolonged grief-related symptoms

PRISMA Checklist

Supplementary Table S1: Search Strategy

PubMed - 818

#1	("Child"[Mesh] OR "Pediatrics"[Mesh] OR "Young Adult"[Mesh] OR "Adolescent"[Mesh] OR "Infant"[Mesh] OR "Pediatric*"[Title/Abstract] OR "paediatric*"[Title/Abstract] OR "Child*"[Title/Abstract] OR juvenile*[Title/Abstract] OR infant*[Title/Abstract] OR adolesc*[Title/Abstract])
#2	("Grief"[Mesh] OR “Prolonged Grief Disorder”[Mesh] OR "Bereavement"[Mesh] OR "Parental Death"[Mesh] OR “grie*”[Title/Abstract] OR “bereav*”[Title/Abstract] OR “Mourn*”[Title/Abstract])
#3	(neoplas*[Title/Abstract] OR cancer*[Title/Abstract] OR tumo*[Title/Abstract] OR malign*[Title/Abstract] OR leukemia*[Title/Abstract] OR lymphoma*[Title/Abstract])

#1 and #2 and #3

Embase - 742

#1	'juvenile'/exp OR 'young adult'/exp OR 'adolescent'/exp OR 'child'/exp OR 'pediatric*':ti,ab OR 'paediatric*':ti,ab OR 'childhood':ti,ab
#2	'grief'/exp OR 'prolonged grief'/exp OR 'complicated grief'/exp OR 'mourning'/exp OR 'bereavement'/exp OR 'parental death'/exp
#3	(neoplas*[Title/Abstract] OR cancer*[Title/Abstract] OR tumo*[Title/Abstract] OR malign*[Title/Abstract] OR leukemia*[Title/Abstract] OR lymphoma*[Title/Abstract])

#1 and #2 and #3

Supplementary Table S2: De-duplication Workflow for Overlapping Cohorts and Final Study Selection

Country	Studies with Potential Overlap	Data Source	Patient Inclusion Period	Population studied	Study Retained for Meta-Analysis (Reason)	Studies Retained for Systematic Review for Prognostic Factors
Sweden	Wallin 2016<br>Eilegård 2013,<br>Sveen 2014,<br>Lövgren 2018	Swedish Childhood Cancer Registry	2000-2007	Sibling (Eilegård 2013, Sveen 2014, Lövgren 2018)<br><br>Parents (Wallin 2016)	Sveen 2014 (Larger population size)	Wallin 2016,<br>Eilegård 2013,<br>Sveen 2014,<br>Lövgren 2018
Sweden	Bylund-Grenklo 2021,<br>Bylund-Grenklo 2016,<br>Beernaert 2017,<br>Birgisdóttir 2023	Multigenerational Register at Statistics Sweden	2000-2003	Parents	Bylund-Grenklo 2016 (Largest proportion and more data on relevant mental health outcomes)	Bylund-Grenklo 2021,<br>Bylund-Grenklo 2016,<br>Beernaert 2017,<br>Birgisdóttir 2023

Supplementary Table S3: Evaluation of the mediating or confounding effect of pre-existing depression in young individuals with prolonged grief-related symptoms

Author	Year	Country	Study population	Key findings†
T. Bylund-Grenklo	2016	Sweden	559 participants with a mean age of 22 years old, were recruited as part of a nationwide population study in Sweden.	Moderate to severe depression was statistically significantly associated with unresolved grief [sons (RR=3.6, 95%CI: 1.4–8.8; daughters (RR=1.8, 95%CI: 1.1–3.1)].
A. Eilegård	2013	Sweden	240 bereaved siblings and 293 non-bereaved counterparts were recruited in a nationwide follow-up study in Sweden.	No statistically significant difference was found for depression between the bereaved and non-bereaved siblings (p=0.946).
M. Lövgren	2018	Sweden	174 cancer-bereaved siblings with a mean age of 24 years old, SD 3.8, were recruited in a nationwide population study in Sweden.	Depression was statistically significantly associated with unresolved grief (RR=1.27, 95%CI: 1.12-1.45).

Abbreviations: SD, standard deviation; RR, Risk ratio; CI, Confidence interval

†Outcomes of interest include logistic or linear regression analysis for any association between pre-existing depression and young individuals with prolonged grief-related symptoms

Supplementary Table S4: Evaluation of the mediating or confounding effect of emotional problems in young individuals with prolonged grief-related symptoms

Author	Year	Country	Study population	Key findings†
T. Bylund-Grenklo	2016	Sweden	559 participants with a mean age of 22 years old, were recruited as part of a nationwide population study in Sweden.	Cancer-bereaved sons with unresolved grief were found to have statistically significantly increased crude risks of emotional numbness (RR=2.6, 95%CI: 1.2-5.6) and self-injuring (RR=2.3, 95%CI: 1.1-4.9).
Weber	2021	Sweden	49 families with a children's mean age of 12.7 years old, SD 4.42, were recruited in a study conducted in Sweden.	For children aged 4 to 11 years, the Emotional Problems subscale was significantly positively correlated with parent-reported symptoms of prolonged grief (p=0.00).<br><br>For adolescents aged 12 to 20 years, the Emotional Problems and Total Problems subscales were significantly positively correlated with parent-reported symptoms of prolonged grief (p<0.001).<br><br>Adolescents' self-reported symptoms of prolonged grief were associated with emotional and conduct problems (p=0.02).

Abbreviations: SD, standard deviation; RR, Risk ratio; CI, Confidence interval

†Outcomes of interest include logistic or linear regression analysis for any association between emotional problems and young individuals with prolonged grief-related symptoms

Supplementary Table S5: Evaluation of the mediating or confounding effect of insomnia in young individuals with prolonged grief-related symptoms

Author	Year	Country	Study population	Key findings†
T. Bylund-Grenklo	2016	Sweden	559 participants with a mean age of 22 years old were recruited as part of a nationwide population study in Sweden.	Insomnia one to three times a week or more was statistically significantly associated with unresolved grief [sons (RR=2.3, 95%CI: 1.3–4.0); daughters (RR=1.7, 95%CI: 1.1–2.7)].
A. Eilegård	2013	Sweden	240 bereaved siblings and 293 non-bereaved counterparts were recruited in a nationwide follow-up study in Sweden.	Insomnia was more prevalent at follow-up among bereaved siblings when compared with non-bereaved peers (p=0.005).

Abbreviations: RR, Risk ratio; CI, Confidence interval

†Outcomes of interest include logistic or linear regression analysis for any association between insomnia and young individuals with prolonged grief-related symptoms

Supplementary Table S6: Evaluation of the mediating or confounding effect of gender in young individuals with prolonged grief-related symptoms

**Author**	**Year**	**Country**	**Study population**	**Key findings†**
Angelhoff	2021	Sweden	22 family dyads with children's mean age of 15.3 years old, SD 2, were recruited as part of a study using the Swedish National Causes of Death Register and the Multi-Generational Register at Statistics Sweden in Sweden.	No statistically significant differences in prolonged grief were found between genders (p>0.05).
T. Bylund-Grenklo	2016	Sweden	559 participants with a mean age of 22 years old were recruited as part of a nationwide population study in Sweden.	Females are more significantly associated with unresolved grief than males (RR=1.3, 95%CI: 1.1–1.6).
O. Rasouli	2022	Norway	99 young adults with a mean age of 22.6 years old, SD 2.3, were recruited in this Norwegian nationwide study.	There is no significant association between gender and grief (p=0.89).

Abbreviations: SD, standard deviation; RR, Risk ratio; CI, Confidence interval

†Outcomes of interest include logistic or linear regression analysis for any association between gender and young individuals with prolonged grief-related symptoms

Supplementary Table S7: Evaluation of the mediating or confounding effect of the duration since loss in young individuals with prolonged grief-related symptoms

Author	Year	Country	Study population	Key findings†
Angelhoff	2021	Sweden	22 family dyads with children's mean age of 15.3 years old, SD 2, were recruited as part of a study using the Swedish National Causes of Death Register and the Multi-Generational Register at Statistics Sweden in Sweden.	Time since death was not significantly correlated with symptoms of prolonged grief (p>0.05).
M. Lövgren	2018	Sweden	174 cancer-bereaved siblings with a mean age of 24 years old, SD 3.8, were recruited in a nationwide population study in Sweden.	Time since death of 2 to 4 years (RR=3.11, 95%CI: 1.34-7.27) and 5 to 7 years (RR=2.6, 95%CI: 1.19-5.96) were significantly associated with unresolved grief.

Abbreviations: SD, standard deviation; RR, Risk ratio; CI, Confidence interval

†Outcomes of interest include logistic or linear regression analysis for any association between duration since loss and young individuals with prolonged grief-related symptoms

Supplementary Table S8: Evaluation of the mediating or confounding effect of self-esteem in young individuals with prolonged grief-related symptoms

**Author**	**Year**	**Country**	**Study population**	**Key findings†**
Angelhoff	2021	Sweden	22 family dyads with children's mean age of 15.3 years old, SD 2, were recruited as part of a study using the Swedish National Causes of Death Register and the Multi-Generational Register at Statistics Sweden in Sweden.	There was a non-significant tendency that high levels of prolonged grief were related to low self-esteem in both groups (adolescents: n=20, p=0.08, parents: n=17, p=0.06).
A. Eilegård	2013	Sweden	240 bereaved siblings and 293 non-bereaved counterparts were recruited in a nationwide follow-up study in Sweden.	Low self-esteem was more prevalent at follow-up among bereaved siblings when compared with non-bereaved peers (p=0.002).

Abbreviations: SD, standard deviation

†Outcomes of interest include logistic or linear regression analysis for any association between self-esteem and young individuals with prolonged grief-related symptoms

Supplementary Table S9: Quality assessment of included cross-sectional studies using the Joanna Brigg's Institute Critical Appraisal tool

Study	1	2	3	4	5	6	7	8
Angelhoff	Y	Y	Y	Y	N	N	Y	Y
Beernaert	Y	Y	Y	N	Y	Y	Y	Y
Birgisdóttir	Y	Y	Y	N	Y	Y	Y	Y
Bylund-Grenklo 2021	Y	Y	Y	Y	Y	Y	Y	Y
Bylund-Grenklo 2016	Y	Y	Y	Y	Y	Y	Y	Y
Eilegård	Y	Y	Y	N	Y	Y	Y	Y
Lundberg	Y	Y	Y	N	Y	Y	Y	Y
Weber	Y	Y	Y	Y	Y	Y	Y	Y
Sveen	Y	Y	Y	Y	Y	Y	Y	Y
Lövgren	Y	Y	Y	N	Y	Y	Y	Y
Rasouli	Y	Y	Y	Y	Y	Y	Y	Y
Wallin	Y	Y	Y	N	Y	Y	Y	Y
Rosenburg	Y	Y	Y	N	Y	Y	Y	Y

Checklist
1. Were the criteria for inclusion in the sample clearly defined?
2. Were the study subjects and the setting described in detail?
3. Was the exposure measured in a valid and reliable way?
4. Were objective, standard criteria used for measurement of the condition?
5. Were confounding factors identified?
6. Were strategies to deal with confounding factors stated?
7. Were the outcomes measured in a valid and reliable way?
8. Was appropriate statistical analysis used?

Legend:
Y – Yes
N – No
U – Unclear
NA – Not applicable

Supplementary Figure S1: Leave-one-out analyses of studies assessing the proportion of young individuals with prolonged grief-related symptoms

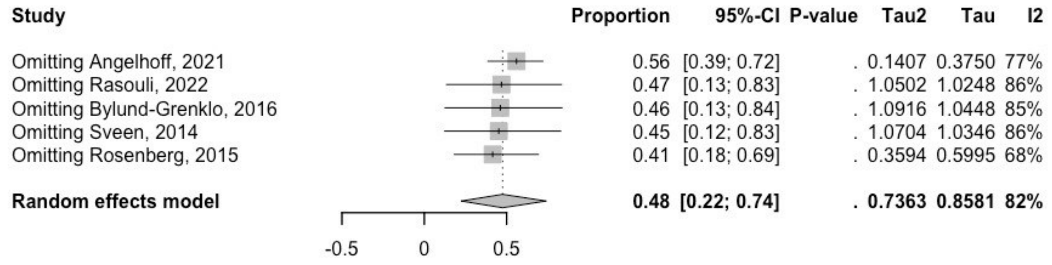

Supplementary Figure S2: Outlier assessment of studies assessing the proportion of young individuals with prolonged grief-related symptoms

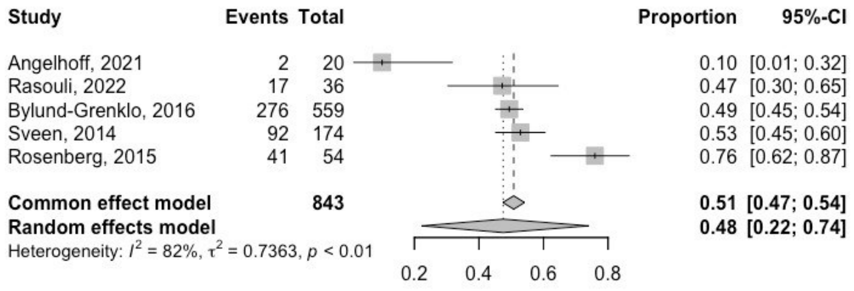

\

PRISMA Checklist

Section and Topic	Item #	Checklist item	Location where item is reported
**TITLE**			
Title	1	Identify the report as a systematic review.	Page 1
**ABSTRACT**			
Abstract	2	See the PRISMA 2020 for Abstracts checklist.	Page 1
**INTRODUCTION**			
Rationale	3	Describe the rationale for the review in the context of existing knowledge.	Page 2
Objectives	4	Provide an explicit statement of the objective(s) or question(s) the review addresses.	Page 2
**METHODS**			
Eligibility criteria	5	Specify the inclusion and exclusion criteria for the review and how studies were grouped for the syntheses.	Page 5
Information sources	6	Specify all databases, registers, websites, organisations, reference lists and other sources searched or consulted to identify studies. Specify the date when each source was last searched or consulted.	Page 3
Search strategy	7	Present the full search strategies for all databases, registers and websites, including any filters and limits used.	Supplementary materials page 2
Selection process	8	Specify the methods used to decide whether a study met the inclusion criteria of the review, including how many reviewers screened each record and each report retrieved, whether they worked independently, and if applicable, details of automation tools used in the process.	Page 5
Data collection process	9	Specify the methods used to collect data from reports, including how many reviewers collected data from each report, whether they worked independently, any processes for obtaining or confirming data from study investigators, and if applicable, details of automation tools used in the process.	Page 5
Data items	10a	List and define all outcomes for which data were sought. Specify whether all results that were compatible with each outcome domain in each study were sought (e.g. for all measures, time points, analyses), and if not, the methods used to decide which results to collect.	Page 2-5
	10b	List and define all other variables for which data were sought (e.g. participant and intervention characteristics, funding sources). Describe any assumptions made about any missing or unclear information.	Page 2-5
Study risk of bias assessment	11	Specify the methods used to assess risk of bias in the included studies, including details of the tool(s) used, how many reviewers assessed each study and whether they worked independently, and if applicable, details of automation tools used in the process.	Page 5
Effect measures	12	Specify for each outcome the effect measure(s) (e.g. risk ratio, mean difference) used in the synthesis or presentation of results.	Page 5
Synthesis methods	13a	Describe the processes used to decide which studies were eligible for each synthesis (e.g. tabulating the study intervention characteristics and comparing against the planned groups for each synthesis (item #5)).	Page 5
	13b	Describe any methods required to prepare the data for presentation or synthesis, such as handling of missing summary statistics, or data conversions.	Page 2-5

Section and Topic	Item #	Checklist item	Location where item is reported
	13c	Describe any methods used to tabulate or visually display results of individual studies and syntheses.	Page 2-5
	13d	Describe any methods used to synthesize results and provide a rationale for the choice(s). If meta-analysis was performed, describe the model(s), method(s) to identify the presence and extent of statistical heterogeneity, and software package(s) used.	Page 5
	13e	Describe any methods used to explore possible causes of heterogeneity among study results (e.g. subgroup analysis, meta-regression).	Page 5
	13f	Describe any sensitivity analyses conducted to assess robustness of the synthesized results.	Page 5
Reporting bias assessment	14	Describe any methods used to assess risk of bias due to missing results in a synthesis (arising from reporting biases).	Page 5
Certainty assessment	15	Describe any methods used to assess certainty (or confidence) in the body of evidence for an outcome.	Page 5
**RESULTS**			
Study selection	16a	Describe the results of the search and selection process, from the number of records identified in the search to the number of studies included in the review, ideally using a flow diagram.	Page 6
	16b	Cite studies that might appear to meet the inclusion criteria, but which were excluded, and explain why they were excluded.	Page 6
Study characteristics	17	Cite each included study and present its characteristics.	Page 3-4
Risk of bias in studies	18	Present assessments of risk of bias for each included study.	Page 9 and Supplementary table 8
Results of individual studies	19	For all outcomes, present, for each study: (a) summary statistics for each group (where appropriate) and (b) an effect estimate and its precision (e.g. confidence/credible interval), ideally using structured tables or plots.	Page 7-9
Results of syntheses	20a	For each synthesis, briefly summarise the characteristics and risk of bias among contributing studies.	Page 7-9
	20b	Present results of all statistical syntheses conducted. If meta-analysis was done, present for each the summary estimate and its precision (e.g. confidence/credible interval) and measures of statistical heterogeneity. If comparing groups, describe the direction of the effect.	Page 7-9
	20c	Present results of all investigations of possible causes of heterogeneity among study results.	Page 7-9
	20d	Present results of all sensitivity analyses conducted to assess the robustness of the synthesized results.	Page 7-9
Reporting biases	21	Present assessments of risk of bias due to missing results (arising from reporting biases) for each synthesis assessed.	Page 9
Certainty of evidence	22	Present assessments of certainty (or confidence) in the body of evidence for each outcome assessed.	Page 7-9
**DISCUSSION**			

Section and Topic	Item #	Checklist item	Location where item is reported
Discussion	23a	Provide a general interpretation of the results in the context of other evidence.	Page 9-12
	23b	Discuss any limitations of the evidence included in the review.	Page 12
	23c	Discuss any limitations of the review processes used.	Page 12
	23d	Discuss implications of the results for practice, policy, and future research.	Page 9-12
**OTHER INFORMATION**			
Registration and protocol	24a	Provide registration information for the review, including register name and registration number, or state that the review was not registered.	Page 2
	24b	Indicate where the review protocol can be accessed, or state that a protocol was not prepared.	Page 2
	24c	Describe and explain any amendments to information provided at registration or in the protocol.	NA
Support	25	Describe sources of financial or non-financial support for the review, and the role of the funders or sponsors in the review.	Page 12-13
Competing interests	26	Declare any competing interests of review authors.	Page 12-13
Availability of data, code and other materials	27	Report which of the following are publicly available and where they can be found: template data collection forms; data extracted from included studies; data used for all analyses; analytic code; any other materials used in the review.	Page 12-13

From: Page MJ, McKenzie JE, Bossuyt PM, Boutron I, Hoffmann TC, Mulrow CD, et al. The PRISMA 2020 statement: an updated guideline for reporting systematic reviews. BMJ 2021;372:n71. doi: 10.1136/bmj.n71

For more information, visit: http://www.prisma-statement.org/
